# Mecanismo causante de Acidente Vascular Cerebral Embólico na Cardiopatia Chagásica Crônica: Disfunção Autonômica, uma Hipótese de Trabalho

**DOI:** 10.36660/abc.20200481

**Published:** 2020-12-01

**Authors:** Roberto Coury Pedrosa

**Affiliations:** 1 Universidade Federal do Rio de Janeiro Rio de JaneiroRJ Brasil Universidade Federal do Rio de Janeiro, Rio de Janeiro, RJ – Brasil

**Keywords:** Acidente Vascular Cerebral, Cardiopatia Chagásica

No Brasil, até o momento, a doença de Chagas (DC) ainda é um grande problema de saúde pública e causa frequente de cardiomiopatia chagásica crônica (CCC) e acidente vascular cerebral (AVC).^1^ Ainda é a principal causa de DALYs — anos de vida ajustados por incapacidade (em 2016, de 141.640), uma medida de perda de saúde devido à carga de doenças fatais e não fatais.^2^ A doença de Chagas está fortemente associada ao AVC embólico associado à doença de Chagas (AVC-DC)^3^ com uma incidência que varia de 0,56 a 2,67 por 100 pessoas/ano.^4 , 5^ Está intimamente relacionado à presença de CCC.^6^ O diagnóstico de DC pode ser estabelecido após ocorrência de AVC em cerca de 40% dos pacientes. Classicamente, a causa de AVC-DC era considerada cardioembólica, com trombo intracardíaco resultante de função ventricular deficiente e arritmia atrial. Os fatores de risco para AVC-DC incluem aneurisma apical, trombo de ventrículo esquerdo (VE), dilatação atrial importante, disfunção sistólica de VE, idade avançada e arritmia atrial^5^. Embora seja verdade que a maioria dos AVC-DC é tromboembólica, outros tipos, incluindo a doença de pequenos vasos, entre outras (aterosclerose e AVC criptogênico), têm sido observadas.^7^ Hipóteses sobre os mecanismos desses AVCs não cardioembólicos incluem a presença de disfunção autonômica na maioria ou em todos os pacientes com CCC em estágios diversos.^8^ Com relação à disfunção autonômica, nosso grupo mostrou que pacientes com CCC, mesmo em estágios iniciais e sem disfunção cardíaca, apresentam sinais de um distúrbio do sistema nervoso parassimpático que se correlacionam significativamente com anormalidades da substância branca subcortical cerebral. Isso foi demonstrado por uma correlação inversa e significativa entre a variabilidade da frequência cardíaca reduzida (avaliada pelo teste de arritmia sinusal respiratória e a presença e o número de hiperintensidades da substância branca vistas em exames de ressonância magnética (RM) craniana. No total, 52% dos nossos pacientes apresentaram hiperintensidades na RM, em comparação com cerca de 13% na população geral. No entanto, não houve correlação com a hemodinâmica cerebral, que foi testada com Doppler transcraniano, incluindo a vasorreatividade avaliada pelo índice de apneia. Com base nesses resultados, sugerimos que um sistema parassimpático funcionando adequadamente proteja o cérebro contra hiperintensidades da substância branca. Especulamos que o desequilíbrio entre os sistemas simpático e parassimpático poderia promover instabilidade elétrica do coração, que poderia contribuir para o AVC-DC não cardioembólico.^9^ Para responder se a disfunção parassimpática é um mediador das consequências neurológicas do CCC ou apenas um fator adjunto, realizamos outro estudo onde não foi encontrada correlação entre a presença de anticorpos circulantes funcionais séricos com atividade β-adrenérgica ou muscarínica e a função do sistema autônomo ou a presença de hiperintensidades da substância branca (observadas na ressonância magnética) em pacientes com CCC. Os anticorpos antirreceptores foram testados em corações isolados de coelhos. Concluímos que a disfunção autonômica é possivelmente um mediador das consequências neurológicas da CCC, aparentemente relacionadas com a promoção de arritmias atriais como epifenômenos de AVC.^10^ Uma característica importante da CCC é o fato de que nem todos os pacientes com fatores de risco para AVC-DC não cardioembólico terão AVC. O complicado desenvolvimento e patologia da doença, juntamente com as complexidades do ciclo de vida do parasita e suas interações com o hospedeiro, dificultam identificar qual grupo de pacientes com CCC é suscetível ao AVC. Embora não haja prova absoluta de que a disfunção autonômica seja sinônimo de AVC-DC não cardioembólico, ou seja, interromper a disfunção autonômica para prevenir AVC-DC não cardioembólico, há um consenso de que a disfunção autonômica cardíaca é necessária para essa ocorrência.

Nesta edição, Freitas et al.,^11^ apresentara um estudo para ajudar a compreender uma apresentação clínica importante na CCC, ou seja, o AVC. Eles chamam a atenção para o mecanismo arrítmico, particularmente a arritmia atrial. A justificativa é que não existe consenso sobre a contribuição dos episódios atriais de alta frequência nesse episódio. Para responder a essa pergunta, os autores realizaram um estudo longitudinal entre 2016 e 2017 para verificar a existência (ou não) de associação entre episódios atriais de alta frequência (EAAF) e AVE em pacientes com CCC crônica. Todos os 67 pacientes chagásicos tinham dispositivos cardíacos eletrônicos implantáveis (DCEIs) (92,5%, marcapassos e 7,5%, cardioversores-desfibriladores implantáveis) para monitorar a atividade atrial. EAAF foi considerado como frequência atrial ≥190 batimentos por minuto e duração ≥6 minutos e os eventos isquêmicos cerebrais foram identificados por exames de tomografia computadorizada (TC) craniana. Os resultados foram resumidos e analisados nos grupos com e sem episódios atriais de alta frequência.

Idade média de 63,6±9,2 anos, acompanhamento de 98±28,8 dias; e 11,9% dos pacientes tinham EAAFs ≥6 min. A TC mostrou eventos isquêmicos cerebrais silenciosos em 16,4% dos pacientes, 63,6% dos quais tinham EAAFs ≥6 min na análise dos DCEIs. Idade avançada [OR 1,12 (IC 95% 1,03–1,21; p<0.009)] e a presença de EAAFs ≥6 minutos [OR 96,2 (IC 95% 9,4–987,4; p<0,001)] foram preditores independentes para eventos isquêmicos. Eles concluíram que os EAAFs detectados pelos DCEIs estavam associados à presença de eventos isquêmicos cerebrais silenciosos em pacientes chagásicos.

A interpretação desses resultados deve levar em consideração o pequeno número de pacientes previamente mencionado pelos autores, fatores de confusão não controlados e a incerteza introduzida pelo grande intervalo de confiança de 95% observado nos pacientes com EAAFs ≥6 minutos. Diferentes estágios de CCC foram possivelmente utilizados, pois há um amplo espectro de manifestações clínicas e patológicas nos pacientes com CCC levando a diferentes perfis de pacientes incluídos nos grupos.^12^ Possivelmente, o estudo se refere a AVC-DC não cardioembólico, uma vez que a fração de ejeção do ventrículo esquerdo (FEVE) e o diâmetro atrial (DA) esquerdo eram normais (apenas 27,3% DA≥40 mm) e não contém nenhuma informação sobre a existência de trombo intracardíaco e aneurisma apical. Uma questão importante é que o nível de AVC-DC não cardioembólico relatado foi muito alto para um acompanhamento médio de apenas 3 meses. Não temos conhecimento de nenhum estudo ou registro que mostre uma incidência tão elevada em pacientes com CCC de idade e características clínicas semelhantes.^4 , 5^ Outra questão importante é a aplicabilidade clínica do estudo, uma vez que seu principal resultado não altera a tomada de decisão em pacientes com CCC. Não há evidências de que os pacientes com AVC-DC não embólico devam ser submetidos a uma investigação diagnóstica diferente de outros pacientes com AVC e agentes antiplaquetários para AVC-DC não cardioembólico. Faltam dados concretos sobre tratamentos eficazes neste subgrupo bastante grande de pacientes. No entanto, é importante entender por que esses pacientes sem disfunção sistólica do ventrículo esquerdo evidente (FEVE 58,5±14,1) pareciam ter aumento da incidência de AVC-DC não embólico. Uma possível explicação é a presença de disfunção autonômica observada em pacientes com CCC não dilatada, que está relacionada à redução dos índices de variabilidade cardíaca vagal. A disfunção cardíaca vagal pode ocorrer mesmo em pacientes com CCC nos estágios iniciais, mas piora à medida que a função ventricular esquerda se deteriora com a progressão da doença^13 , 14^ e um sistema parassimpático funcionando adequadamente para proteger o cérebro contra AVC-DC não cardioembólico.

Embora diversos aspectos do AVC-DC não embólico na CCC tenham sido elucidados nas últimas décadas, importantes questões permanecem sem solução quanto aos seus mecanismos. Embora alguns estudos na área pré-clínica tenham documentado o papel dos distúrbios da modulação autonômica no AVC-DC não cardioembólico, trata-se ainda de hipóteses preliminares e os aspectos translacionais desses estudos não foram totalmente alcançados. Um melhor entendimento da função da modulação autonômica na gravidade clínica do AVC-DC não embólico na CCC é uma área importante de pesquisa para estudos futuros.
