# Supplementary material for: Mecanismo causante de Acidente Vascular Cerebral Embólico na Cardiopatia Chagásica Crônica: Disfunção Autonômica, uma Hipótese de Trabalho
Source: Arq Bras Cardiol. 2020 Dec 1;115(6):1080–1. [Article in Portuguese] doi: 10.36660/abc.20200481 (PMC8133738; doi:10.36660/abc.20200481)
Supplement: Translation

# Translation: Causing Mechanisms of Embolic Strokes in Chagas Heart Disease: Autonomic Dysfunction, a Working Hypothesis

**Authors:** Roberto Coury Pedrosa

**Affiliations:** 1 Brazil Universidade Federal do Rio de Janeiro, Rio de Janeiro, RJ – Brazil

**Keywords:** Stroke, Chagas Heart Disease

In Brazil, to date, Chagas disease (Chd) is still a major public health problem and a frequent cause of chronic Chagas cardiomyopathy (CCC) and stroke.^1^It is still the leading cause of DALYs — disability-adjusted life-years (in 2016, of 141,640), a measure of health loss due to fatal and non-fatal disease burden.^2^Chd is strongly associated with Chagas association-embolic strokes (CAS)^3^with an incidence ranging from 0.56 to 2.67 per 100 person/year.^4 , 5^It is closely related to the presence of CCC.^6^Diagnosis of Chd may be established after stroke presentation in around 40% of patients. Classically, the cause of CAS was thought to be cardioembolic, with intracardiac thrombus resulting from poor ventricular function and atrial arrhythmia. Risk factors for CAS include apical aneurysm, left ventricular (LV) thrombus, severe atrial dilation, LV systolic dysfunction, older age, and atrial arrhythmia.^5^Although it remains true that the majority of CAS is thromboembolic, other types, including small vessel disease, among others (i.e. vessel atherosclerosis and cryptogenic stroke), have been observed.^7^Hypotheses on the mechanisms of these noncardioembolic strokes include the presence of autonomic dysfunction in most or all patients with CCC in various stages.^8^Concerning autonomic dysfunction, our group showed that CCC patients, even in the early stages and without cardiac dysfunction, present signs of a parasympathetic nervous system disorder that correlate significantly with subcortical white matter abnormalities of the brain. This was demonstrated by an inverse and significant correlation between reduced cardiac frequency variability (evaluated by the respiratory sinus arrhythmia test and the presence and number of white matter hyperintensities seen on brain magnetic resonance imaging (MRI). A total of 52% of our patients revealed hyperintensities on MRI, compared with about 13% in the general population. However, there was no correlation with cerebral hemodynamics, which was tested with a transcranial Doppler, including vasoreactivity evaluated by the breath-holding index. Based on these results, we suggested that a properly functioning parasympathetic system protect the brain against white matter hyperintensities. We speculated that imbalance between sympathetic and parasympathetic systems could promote electrical instability of the heart that could contribute to noncardioembolic CAS.^9^To answer whether the parasympathetic dysfunction is a mediator of neurologic consequences of CCC or just a bystander, we conducted another study where we found no correlation between the presence of serum functional circulating antibodies with β-adrenergic or muscarinic activity and the autonomic system function or the presence of white matter hyperintensities (seen on MRI) in CCC patients. The antireceptor antibodies were tested in rabbit isolated hearts. We concluded that autonomic dysfunction is possibly a mediator of neurologic consequences of CCC apparently related to promote atrial arrhythmias as epiphenomena of stroke.^10^An important feature of CCC is the fact that not all patient with risk factors for noncardioembolic CAS will have stroke. The complicated development and pathology of the disease, coupled with the complexities of the parasite life cycle and its interactions with the host, make it a very difficult task to identify which group of patients with CCC is susceptible to stroke. Although there is no absolute proof that autonomic dysfunction is synonymous with noncardioembolic CAS, i.e., halting autonomic dysfunction to prevent noncardioembolic CAS, there is a consensus that cardiac autonomic dysfunction is needed for this event.

In this edition, Freitas et al.^11^presented a study to help understand an important clinical presentation in CCC, i.e. stroke. They draw attention to the arrhythmic mechanism, particularly atrial arrhythmia. The justification is that a consensus is lacking about the contribution of atrial high-rate episodes in this event. To answer this question, the authors performed a longitudinal study between 2016 and 2017 to check the existence (or not) of an association between high-rate atrial episodes (HRAE) and stroke in patients with chronic CCC. All 67 Chagas patients had implantable electronic cardiac devices (IECDs) (92.5% had pacemakers and 7.5% had implantable cardioverter-defibrillators) to monitor atrial activity. HRAE was considered as atrial frequency ≥190 beats per minute and duration ≥6 minutes and cerebral ischemic events were identified by brain computed tomography (CT) scans. The results were summarized and analyzed in the groups with and without atrial high-rate episodes.

Mean age of 63.6±9.2 years, follow-up for 98±28.8 days; and 11.9% of the patients had HRAEs ≥6 min. CT showed silent brain ischemic events in 16.4% of the patients, 63.6% of whom had HRAEs ≥6 min in the analysis of IECDs. Advanced age [OR 1.12 (95% CI 1.03–1.21; p<0.009] and the presence of HRAEs ≥6 minutes [OR 96.2 (95% CI 9.4–987.4; p<0.001)] were independent predictors for ischemic events. They concluded that HRAEs detected by IECDs were associated with the presence of silent brain ischemic events in Chagas patients.

The interpretation of these results should consider the small number of patients previously mentioned by the authors, uncontrolled confounding factors and the uncertainty introduced by the large 95% confidence interval observed in the patients with HRAEs ≥6 minutes. Different stages of CCC was possibly used since there is a wide spectrum of clinical and pathological manifestations in CCC patients leading to different profiles of patients included in the groups.^12^Possibly, the study refers to noncardioembolic CAS, since left ventricular ejection fraction (LVEF) and left atrial (LA) diameter were normal (only 27.3% LA ≥40 mm) and it does not contain any information about existence of intracardiac thrombus and apical aneurysm. An important question is that the level of noncardioembolic CAS reported was very high for a mean follow-up of only 3 months. We are not aware of any study or registries that show such a high incidence in CCC patients of similar age and clinical characteristics.^4 , 5^Another important question is the clinical applicability of the study since its main result does not change decision-making in CCC patients. There is no evidence that patients with non-embolic CAS should undergo a different diagnostic workup than other stroke patients and antiplatelet agents for noncardioembolic CAS. Actual data on effective treatments are lacking in this rather large subgroup of patients. However, it is important to understand why such patients without overt left ventricular systolic dysfunction (LVEF 58.5±14.1) seemed to have increased non-embolic CAS. One putative explanation is the presence of autonomic dysfunction observed in nondilated CCC patients, which is related to reduced vagal heart variability indexes. Vagal heart dysfunction can occur even in CCC patients in the early stages, but it worsens as the left ventricular function deteriorates with disease progression^13 , 14^and a properly functioning parasympathetic system to protect the brain against noncardioembolic CAS.

Although several aspects of non-embolic CAS in CCC have been elucidated in the last decades, important questions remain unresolved as to its mechanisms. While some studies in the pre-clinical area have documented the role of autonomic modulation disorders in noncardioembolic CAS, this still represents a preliminary hypothesis and the translational aspects of these studies have not been fully achieved. A better understanding of the function of autonomic modulation in the clinical severity of non-embolic CAS in CCC is an important area of research for future studies.
